# Activation of α7 Nicotinic Acetylcholine Receptor Upregulates HLA-DR and Macrophage Receptors: Potential Role in Adaptive Immunity and in Preventing Immunosuppression

**DOI:** 10.3390/biom10040507

**Published:** 2020-03-27

**Authors:** Andrei E. Siniavin, Maria A. Streltsova, Denis S. Kudryavtsev, Irina V. Shelukhina, Yuri N. Utkin, Victor I. Tsetlin

**Affiliations:** 1 Department of Molecular Neuroimmune Signalling, Shemyakin-Ovchinnikov Institute of Bioorganic Chemistry, Russian Academy of Sciences, Moscow 117997, Russia; kudryavtsevden@gmail.com (D.S.K.); ner-neri@ya.ru (I.V.S.); yutkin51@gmail.com (Y.N.U.); victortsetlin3f@gmail.com (V.I.T.); 2 N.F. Gamaleya National Research Center for Epidemiology and Microbiology, Ministry of Health of the Russian Federation, Moscow 123098, Russia; 3 Department of Immunology, Shemyakin-Ovchinnikov Institute of Bioorganic Chemistry, Russian Academy of Sciences, Moscow 117997, Russia; mstreltsova@mail.ru; 4 Institute of Engineering Physics for Biomedicine, National Research Nuclear University, Moscow 115409, Russia

**Keywords:** α7 nAChR, macrophages, adaptive immunity, innate immunity, immunosuppression, inflammation

## Abstract

Immune response during sepsis is characterized by hyper-inflammation followed by immunosuppression. The crucial role of macrophages is well-known for both septic stages, since they are involved in immune homeostasis and inflammation, their dysfunction being implicated in immunosuppression. The cholinergic anti-inflammatory pathway mediated by macrophage α7 nicotinic acetylcholine receptor (nAChR) represents possible drug target. Although α7 nAChR activation on macrophages reduces the production of proinflammatory cytokines, the role of these receptors in immunological changes at the cellular level is not fully understood. Using α7 nAChR selective agonist PNU 282,987, we investigated the influence of α7 nAChR activation on the expression of cytokines and, for the first time, of the macrophage membrane markers: cluster of differentiation 14 (CD14), human leukocyte antigen-DR (HLA-DR), CD11b, and CD54. Application of PNU 282,987 to THP-1Mϕ (THP-1 derived macrophages) cells led to inward ion currents and Ca^2+^ increase in cytoplasm showing the presence of functionally active α7 nAChR. Production of cytokines tumor necrosis factor-α (TNF-α), interleukin (IL)-6, and IL-10 was estimated in classically activated macrophages (M1) and treatment with PNU 282,987 diminished IL-10 expression. α7 nAChR activation on THP-1Mϕ, THP-1M1, and monocyte-derived macrophages (MDMs) increased the expression of HLA-DR, CD54, and CD11b molecules, but decreased CD14 receptor expression, these effects being blocked by alpha (α)-bungarotoxin. Thus, PNU 282,987 enhances the macrophage-mediated immunity via α7 nAChR by regulating expression of their membrane receptors and of cytokines, both playing an important role in preventing immunosuppressive states.

## 1. Introduction

Nicotinic acetylcholine receptors (nAChRs) are cationic channels belonging to the Cys-loop receptor family. They are involved in a wide variety of physiological processes, including learning, memory, sensory, and neuromuscular signaling [1]. α7 nAChR is a homopentameric receptor mostly expressed in the central nervous system [2], but also found on such immune system cells as lymphocytes, monocytes, and macrophages [3]. Activation of macrophages with nicotine or acetylcholine stimulates the cholinergic anti-inflammatory pathway (CAP). The role of α7 nAChR is important in the CAP-mediated downregulation of proinflammatory responses. Exposure of lipopolysaccharide (LPS)-activated human macrophages to acetylcholine resulted in a dose-dependent inhibition of such proinflammatory cytokines as tumor necrosis factor-α (TNF-α), interleukin 6 (IL-6), and IL-1β [4], involved in pathological processes of endotoxemia and sepsis [5]. Suppression of cytokine production through α7 nAChR includes the following signaling pathways: inhibition of nuclear factor kappa-light-chain-enhancer of activated B cells (NF-kB) translocation and the inhibitor of kappa-B (I-kB) phosphorylation [6], activation of JAK2-STAT3 signaling [7] and inhibition of the toll-like receptor 4 (TLR4) receptor expression [8]. However, the role of α7 nAChR in the regulation of innate immune cell receptors and their functions in the inflammatory reactions is not yet clear.

Macrophages and monocytes play a central role in the pathogenesis of sepsis. Sepsis manifests itself as a persistent inflammatory response to a bacterial infection, which ultimately can lead to multiple organ dysfunction [9]. This response is triggered by such bacterial toxins as endotoxin of gram-negative organisms (LPS) and a complex of lipoteichoic acid and peptidoglycan of gram-positive bacteria. Cluster of differentiation 14 (CD14) is a high affinity membrane binding protein for LPS. In sepsis caused by gram-negative bacteria, binding of LPS to the CD14 membrane receptor leads to excessive activation of monocytes and macrophages [10]. LPS stimulation also causes activation of NF-kB and p38 mitogen-activated protein kinase (p38 MAPK) [11]. Activated monocytes and macrophages release large amounts of TNF-α, the main mediator of sepsis. In addition to TNF-α, monocytes and macrophages produce other proinflammatory mediators, including IL-1β and IL-6, eicosanoids, reactive oxygen species (ROS), platelet-activating factor, and nitric oxide (NO) [12].

Sepsis affects the immune system, directly altering the lifespan, production, and function of the effector cells responsible for homeostasis. The persistent and simultaneously inflammatory and anti-inflammatory states caused by dysfunctional innate and suppressed adaptive immunity, together lead to persistent organ failure and death of the patient [13]. The expression of the major histocompatibility complex of class II (MHC II; HLA-DR) on monocytes and macrophages is important for effective interaction with T-lymphocytes, especially when presenting antigens. In addition, cell adhesion molecules and the complement system receptors, such as CD54 and CD11b, are necessary for the effective activation of various T-cell populations and for the elimination of pathogens [14,15]. A reduced expression of HLA-DR molecule on macrophages is a hallmark of sepsis and of Systemic Inflammatory Response Syndrome (SIRS) [16], and also is observed after LPS injection to healthy volunteers [17]. Moreover, the degree of HLA-DR expression on monocytes and macrophages in patients with established sepsis is prognostically significant and is a marker of immune dysfunction [18], a reduced expression being more pronounced in patients with superinfection. A decreased expression of MHC class II is commonly described in sepsis, the lowest level of HLA-DR expression being observed among septic patients with a high mortality rate [19]. A low expression of MHC class II leads to an altered ability to present antigen and to an increased risk of infectious complications in patients with sepsis after various injuries [20,21]. Low HLA-DR expression is associated with an increased risk of secondary bacterial infections [22], probably due to a less powerful presentation of the antigen, precluding the creation of an effective adaptive immunity [23].

In this article, for the first time we have studied the influence of the selective α7 nAChR agonist PNU 282,987 on the expression of membrane receptors, namely the LPS-binding receptor CD14, HLA-DR molecules, as well as on the production of cytokines during the macrophages maturation and their classical activation. We found that activation of α7 nAChR on macrophages affects the expression of membrane proteins, such as HLA-DR, CD14, CD11b, and CD54, and also induces changes in the IL-10 production, all being involved in immunosuppression and hyperinflammation during sepsis. Therefore, the activation of the α7 nAChRs and, thus, regulating both the HLA-DR expression and IL-10 production may be a way to prevent the immune dysfunction.

## 2. Materials and Methods

### 2.1. Reagents

LPS (*Escherichia coli* O111:B4), Phorbol 12-myristate 13-acetate (PMA), Poly-L-lysine hydrochloride were purchased from Sigma-Aldrich (St. Louis, MO, USA, country). Fluo-4AM, Probenecid, human Granulocyte-Macrophage Colony-stimulating Factor (GM-CSF), and alpha (α)-bungarotoxin Alexa Fluor 647 conjugate were from ThermoFisher Scientific (Waltham, MA, USA). Ficoll-Paque PLUS was from GE Healthcare (Pittsburgh, PA, USA). Interferon-γ (IFN-γ) was from PeproTech (Rocky Hill, NJ, USA). The following monoclonal antibodies (mAbs) against human molecules: fluorescein isothiocyanate fluorescein isothiocyanate (FITC)-anti-HLA-DR (clone LN3), phycoerythrin (PE)-anti-CD14 (clone HCD14), Alexa Fluor 488-anti-CD54 (clone HCD54), phycoerythrin-cyanine 7 (PE/Cy7)-anti-CD11b (clone ICRF44) and isotype-matched negative controls mAbs were obtained from Sony Biotechnology (San Jose, CA, USA). PNU 282,987, PNU 120,596, nicotine, (±)-epibatidine, methyllycaconitine citrate, dihydro-β-erythroidine hydrobromide, and α-bungarotoxin (α-bgt) were obtained from Tocris (R&D Systems, Minneapolis, MN, USA).

### 2.2. Cell Line and Macrophages Preparation

THP-1 (human monocytic cell line, ATCC, TIB-202) were maintained in complete RPMI 1640 culture medium, supplemented with 10% of heat inactivated fetal bovine serum (FBS), 2 mM L-glutamine, 50 IU/mL penicillin, and 50 µg/mL streptomycin (all from Gibco BRL Life Technologies, Waltham, MA, USA) in a humidified atmosphere of 5% CO_2_ at 37 °C.

This study was carried out in accordance with the Declaration of Helsinki. Blood samples were obtained from healthy volunteers, who gave written informed consent prior to the study in accordance with the recommendations of the local ethics committee of Pirogov Russian National Research Medical University (protocol #169 of the ethics committee meeting, 20.11.2017). Peripheral blood mononuclear cells (PBMCs) were separated by Ficoll-Paque PLUS density gradient centrifugation. PBMCs were placed in a sterile Petri dish and incubated at 37 °C for 2 h. Unattached cells were then removed by washing with PBS, and substituting with fresh complete RPMI 1640 medium. To generate monocyte-derived macrophages (MDMs), 50 ng/mL GM-CSF was added to the isolated monocytes and cultured for 6 days to differentiate them into non-polarized MDMs.

THP-1 monocytes were differentiated into macrophages by 24 h incubation with 100 nM PMA, followed by 48 h incubation in complete RPMI 1640 medium. M1 (classical) polarization was achieved by supplementation with IFN-γ (20 ng/mL) and LPS *E. coli* (100 ng/mL) for 48 h.

For experiments, THP-1 macrophages (THP-1Mϕ and THP-1M1) or human MDMs were cultured in 12-well plates at 5×10^5^ cells/well. To determine the effect of α7 nAChRs agonists on the expression of membrane proteins CD14, HLA-DR, CD11b, and CD54, cells were treated with PNU 282,987 (1 µM) and/or α-bungarotoxin (10 µM) for 48 h.

### 2.3. Single-Cell Ca^2+^ Imaging

Cells transferred to poly-L-lysine covered glass slips were incubated for 2 h at 37 °C. The cells adhered to glass were further incubated with Fluo-4AM given at 2 mM and organic anion transporter inhibitor probenecid at 1.25 mM for 1 h at room temperature. After incubation, cells were washed out with extracellular solution (140 mM NaCl, 2 mM CaCl_2_, 2.8 mM KCl, 4 mM MgCl_2_, 20 mM HEPES, 10 mM glucose; pH 7.4). The last wash-out was supplied with 10 µM PNU 120,596 (an α7 nAChR positive allosteric modulator), and, then, Fluo-4 was excited at 485 nm and fluorescence registered at 535 nm. The measurements were performed on Olympus (Japan) epifluorescence microscope with a CAM-XM10 charge-coupled device (CCD). CellA Imaging Software (Olympus Soft Imaging Solutions GmbH, Germany) was used to record video further analyzed with ImageJ. PNU 282,987 (1 µM) was added directly to the cells preparations. The α7 nAChR inhibition was achieved by 10 µM α-bungarotoxin application 5 min before adding the agonist PNU 282,987. The calcium rise was measured relative to the fluorescence base level of each cell.

### 2.4. Fluorescence-Activated Cell Sorting (FACS) Analysis

To study the surface marker expression in THP-1 macrophages or MDMs, the following mAbs were used: HLA-DR-FITC (clone LN3), CD14-PE (clone HCD14), CD54-Alexa Fluor 488 (clone HCD54), and CD11b-PE/Cy7 (clone ICRF44). Cells were detached with cell dissociation solution (non-enzymatic, Sigma-Aldrich (St. Louis, MO, USA)) and stained with fluorescently-labeled mAbs for 30 min on ice in PBS staining buffer (containing 0.5% bovine serum albumin (BSA) and 0.01% sodium azide). After washing twice with PBS staining buffer by centrifugation at 1200 rpm for 7 min at 4 °C, samples were analyzed using a FACSCalibur flow cytometer (BD Biosciences, San Jose, CA, USA) equipped with the 488- and 640-nm lasers, analyzing 100,000 events. Data were processed using FlowJo software 10.0.8. (Three Star Inc., Ashland, OR, USA). THP-1 macrophages were gated in a logarithmic side scatter (SSC) vs. forward scatter (FSC), while primary macrophages were gated in a linear SSC vs. FSC. Dot plots represented as FSC to fluorescence. Data on histograms were acquired in log mode. 

### 2.5. α-Bungarotoxin Binding Assay

To evaluate the expression of α7 nAChRs on the cell membrane THP-1 macrophages and MDMs, cells were detached with cell dissociation solution and stained with 100 nM Alexa Fluor 647-labeled α-bungarotoxin in PBS staining buffer at room temperature for 1 h. After that, the cells were washed twice with PBS staining buffer and analyzed using BD FACSCalibur flow cytometer. Data were processed using FlowJo software 10.0.8. The autofluorescence of THP-1 macrophages and MDMs was subtracted from the fluorescence intensity values of the stained samples. Fifty thousand events were analyzed for each sample and the fluorescence intensity of cells was analyzed from the geometrical mean of fluoresce.

### 2.6. Whole-Cell Patch Clamp

Cells were cultured on glass and transferred to the extracellular solution (140 mM NaCl, 2 mM CaCl_2_, 2.8 mM KCl, 4 mM MgCl_2_, 20 mM HEPES, 10 mM glucose; pH 7.4) prior to the experiments. Whole-cell patch clamp was performed using HEKA amplifier (HEKA Elektronic, Germany). Capillaries were manufactured on the Narishige puller and filled with internal solution (140 mM CsCl, 6 mM CaCl_2_, 2 mM MgCl_2_, 2 mM MgATP, 0.4 mM NaGTP, 10 mM HEPES/CsOH, 20 mM BAPTA/KOH; pH 7.3). Microelectrodes had 6-8 MOhm resistance observed in real time with 5 mV 5 ms test pulse, cells were clamped at –40 mV, using uncompensated fast capacitance and 10 kHz filter. PNU 282,987 or nicotine at 1 µM along with or without 10 µM PNU 120,596 were applied via Fast Step (Warner Instrument, USA) at the flow rate of about 1 mL/min. GOhm seal formation was stimulated with pre-setting of –10 mV pipette potential in cell-attached mode. After the plasma membrane rupture on-cell settings were applied. Currents were monitored and analyzed through Patchmaster software (HEKA Elektronic, Germany). 

### 2.7. ELISA

THP-1 monocytes were differentiated into macrophages as described in Section 2.2. After differentiation, the media were changed for fresh media with different concentration of PNU 282,987, a selective agonist of α7 receptor. The inflammation inductor, LPS *E.coli* (500 µg/mL) was added to THP-1 macrophages. PNU 282,987 was added 30 min before LPS application. Supernatants were collected 24 h post-treatments and stored at −80 °C for further analysis. TNF-α, IL-6 and IL-10 released from THP-1 macrophages to the culture medium were quantified by ELISA kits according to the manufacturer’s instructions (Vektor-Best, Russia).

### 2.8. Quantitative Real-Time PCR (q-PCR)

Total RNA was isolated from THP-1 macrophages and MDMs using the ExtractRNA reagent (Eurogen, Russia) according to the manufacturer’s instruction. First strand complementary DNA (cDNA) synthesis was performed using Maxima H Minus First Strand cDNA Synthesis Kit with dsDNAse from ThermoFisher Scientific (Waltham, MA, USA) according to the manufacturer’s protocol, using a thermo cycler (BioRad) at 37 °C for 2 min, 25 °C for 10 min, 50 °C for 30 min, and 85 °C for 5 min. The cDNA was subject to qRT-PCR on a CFX96 Real-Time PCR Detection System (Bio-Rad, CA, USA) using PowerUP SYBR Green Master Mix (ThermoFisher Scientific) under the following conditions: 95 °C for 3 min, 95 °C for 15 s, 55 °C for 15 s, 72 °C for 20 s (40 cycles). After the reaction was complete, specificity was verified by melting curve analysis. Quantification was performed by normalizing the Ct (cycle threshold) values of each sample to human β-actin. The sequences of the PCR primers used are given in Table 1.

### 2.9. Data and Statistical Analysis

Data were expressed as mean ± SEM or MFI ± SEM (MFI-geometrical mean fluorescence intensity) for an indicated number of independent experiments. The values of MFI for macrophage membrane receptors expressions in non-treated cells were taken as 100%. The changes in the expression of macrophage receptors were calculated from the MFI values obtained for untreated control cells. Statistical analysis using Student’s *t*-test and one-way ANOVA tests with Tukey post hoc test (for three or more experimental groups) were performed using GraphPad Prism version 6 (GraphPad Software Inc., La Jolla, CA, USA). The differences were considered statistically significant at *p* < 0.05.

## 3. Results

### 3.1. Expression of nAChR Subunits in THP-1Mϕ and MDMs

**Figure 1:**
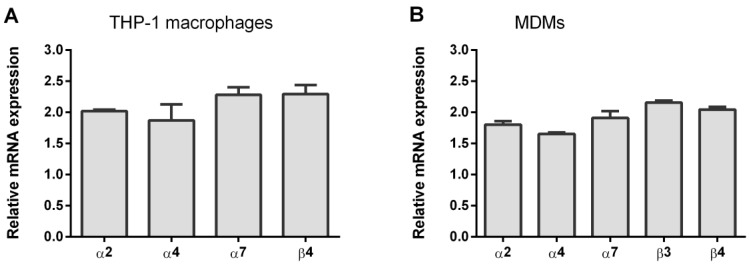
Relative expression of nicotinic acetylcholine receptor (nAChR) subunits transcripts (**A**) human monocytic cell line macrophages (THP-1Mϕ) and (**B**) monocyte-derived macrophages (MDMs) examined by qRT-PCR. Data are shown as relative expression ± SEM, normalized to the endogenous β-actin expression. 3.2. Expression of α7 nAChR on the surface of THP-1Mϕ and MDMs.

Quantitative PCR was performed on first-strand cDNA that was prepared from the THP-1 macrophages or MDMs (Figure 1). Using gene-specific primers, the abundance of mRNA for individual nAChR subunits was determined. The muscle-type nAChR subunit transcript α1 were either undetectable or detected in THP-1ϕ and MDMs. In addition, several neuronal nAChR subunits transcript (α3, α9, β2) were not present. All other human nAChR subunit transcripts (α2, α4, α7, β3, β4) were detected at similar levels.

**Figure 2:**
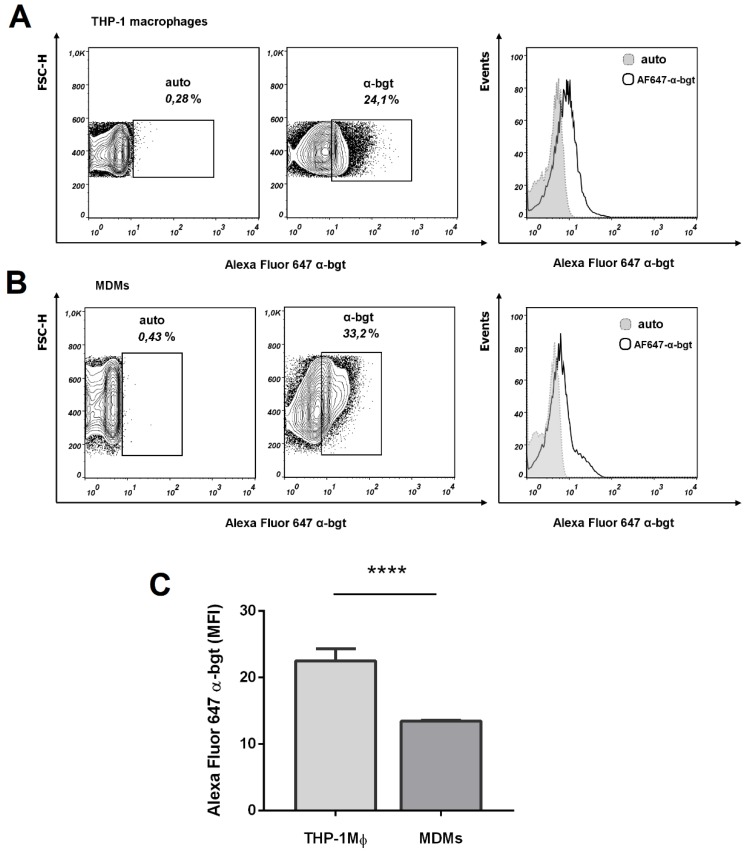
Expression of α7 nAChR on the THP-1Mϕ and MDMs cell surface. THP-1 monocyte or primary human monocytes were differentiated into macrophages (THP-1Mϕ and MDMs, respectively) and stained with Alexa Fluor 647-labeled alpha (α)-bungarotoxin (AF647-α-bgt). Cell staining was analyzed by flow cytometry. (**A**) α7 Receptor expression on the THP-1Mϕ or (**B**) MDMs cells is shown. Gray dotted histograms—autofluorescence of cells; black lined histograms—cells stained with AF647-α-bgt. (**C**) Intensity of AF647-α-bgt staining on THP-1Mϕ and MDMs. Data obtained from three independent experiments performed with MDMs from different donors. Student’s *t*-test: *** *p* < 0.001

Surface expression of α7 nAChR on macrophages obtained after the differentiation of THP-1 cells (THP-1Mϕ) and primary monocytes (MDMs) was analyzed using α7 nAChR antagonist Alexa Fluor 647-labeled α-bgt (AF647-α-bgt). According to flow cytometry results, AF647-α-bgt binding was clearly detected on both THP-1Mϕ (Figure 2A) and MDMs (Figure 2B). Higher fluorescence per cell was observed on THP-1Mϕ (MFI AF647-α-bgt binding on THP-1Mϕ vs. MDMs: 22.5 ± 1.8 vs. 13.4 ± 0.1, **** *p* < 0.0001; Figure 2C). Thus, THP-1Mϕ and MDMs expressed α7 nAChR on their surface, with a higher expression on THP-1Mϕ.

### 3.2. Functional Expression of α7 nAChRs in THP-1 Macrophages (THP-1Mϕ)

**Figure 3:**
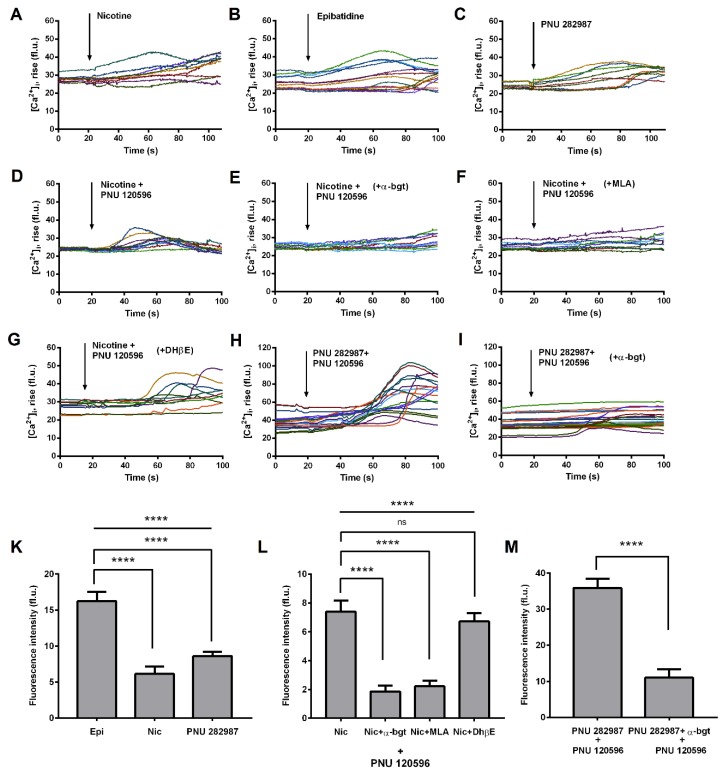
Single-cell Ca^2+^ imaging in THP-1Mϕ. Macrophages obtained from THP-1 cells were loaded with calcium indicator Fluo-4 AM and analyzed using an epifluorescence microscope, as described in the materials and methods. Traces of changes in [Ca^2+^]i in cells over a 100 s are shown. Data were obtained before (0 s) and after (100 s) exposure of the cells to the selective α7 receptor agonist, which corresponds to the time at which the maximum increase in [Ca^2+^]i was observed. The maximum response was observed no later than 80 s; (**A**) 100 µM nicotine, (**B**) 10 µM epibatidine (Epi), (**C**) 1 µM PNU 282,987, (**D**) 100 µM nicotine and 10µM PNU 120,596, (**H**) 1 µM PNU 282,987, and 10 µM PNU 120,596 were applied to the cells; 100 µM nicotine and 10 µM PNU 120,596 were applied to the cells after 20 min pre-incubation with (**E**) 10 µM α-bgt, (**F**) 1 µM methyllycaconitine (MLA), and (**G**) 50 µM dihydro-β-erythroidine (DhβE); (**I**) 1 µM PNU 282,987 and 10 µM PNU 120,596 were applied to the cells after 20 min pre-incubation with 10 µM α-bgt. Each curve represents changes in intracellular Ca^2+^ concentration in one cell. The arrow indicates the time of application of the agonist. (**K**–**M**) The columns are normalized cell response amplitudes expressed as mean ± SEM of the relative fluorescence intensity of each cell (measured at the maximum value minus baseline fluorescence). At least 30–50 cells/condition were analyzed. Fl.u.–arbitrary fluorescent units. One-way ANOVA tests with Turkey post hoc test: **** *p* < 0.0001.

There is much evidence for the presence of α7 nAChRs in the human MDMs [3,27,28,29], while here we analyzed functional expression of these receptors in macrophages obtained after differentiation of the THP-1 cells. Calcium imaging is preferable to measure α7 nAChRs activity in macrophages, since most of previous studies failed to find evidence of their ion channel activity in such cells [30,31,32], referring to their metabotropic rather than ionotropic intracellular signaling [33,34]. Application of non-selective nAChRs agonists nicotine (100 µM) led to calcium rise in some cells (Figure 3A,K). This effect became more prominent after application of more potent agonist epibatidine (Epi, 10 μM, Figure 3B,K) and α7 nAChR selective agonist PNU 282,987 (1 µM, Figure 3C,K). It is very hard or even impossible to detect α7 nAChR-mediated calcium response in neurons and neuroblastoma cells in the absence of a selective positive α7 nAChR allosteric modulator PNU 120,596, decreasing the extremely high rate of receptor desensitization [35,36,37]. Applying PNU 120,596 (10µM) to THP-1ϕ cells, we observed a great increase in the number of nicotine-responsive cells and in the corresponding calcium rise amplitudes (Figure 3D,L). Preincubation of THP-1ϕ cells with 10 μM α-bgt (Figure 3E,L) and 1 μM methyllycaconitine (MLA, Figure 3F,L), but not with 50 µM dihydro-β-erythroidine (DhβE, Figure 3G,L) almost completely abolished the observed calcium rise. Co-application of α7 nAChR selective agonist (1 μM PNU 282,987) and positive allosteric modulator (10 μM PNU 120,596) to THP-1ϕ cells led to calcium rises of the highest amplitude (Figure 3H,M), which were significantly inhibited with 10 μM α-bgt.

**Figure 4:**
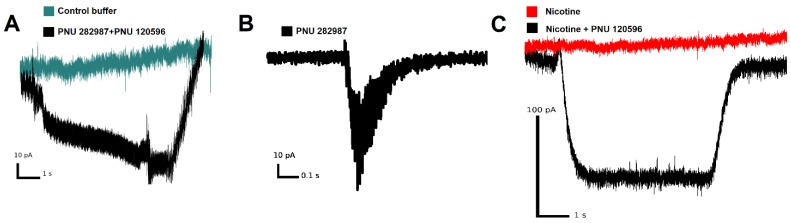
Patch-clamp experiments on THP-1Mϕ. Representative traces for (**A**) buffer (green line) or 1 µM PNU 282,987 with 10 µM PNU 120,596 (black line), (**B**) 1 µM PNU 282,987, and (**C**) 10 µM nicotine (red line) or 10 µM nicotine with 10 µM PNU 120,596 (black line), which have been applied to the cells.

Patch-clamp electrophysiology experiments have demonstrated that α7 nAChR expressed on the THP-1Mϕ is functioning as an ion channel. Figure 4A shows a typical current trace recorded from THP-1Mϕ under simultaneous application of 1 µM PNU 282,987 and 10 µM PNU 120,596. Positive allosteric modulator PNU 120,596 simplifies ion current registration because it removes fast receptor desensitization [38], which is characteristic for α7 nAChR. Five out of twenty-three tested THP-1Mϕ cells have shown ion currents upon application of either PNU 282,987 (with or without PNU 120,596, Figure 4A,B) or of nicotine supplemented with PNU 120,596 (Figure 4C). No cells showing currents when nicotine as such was applied have been detected (Figure 4C).

### 3.3. Activation of α7 nAChRs with PNU 282,987 Regulates the Expression of HLA-DR, CD14, and CD54, CD11b in THP-1Mϕ, THP-1M1, and MDMs

**Figure 5:**
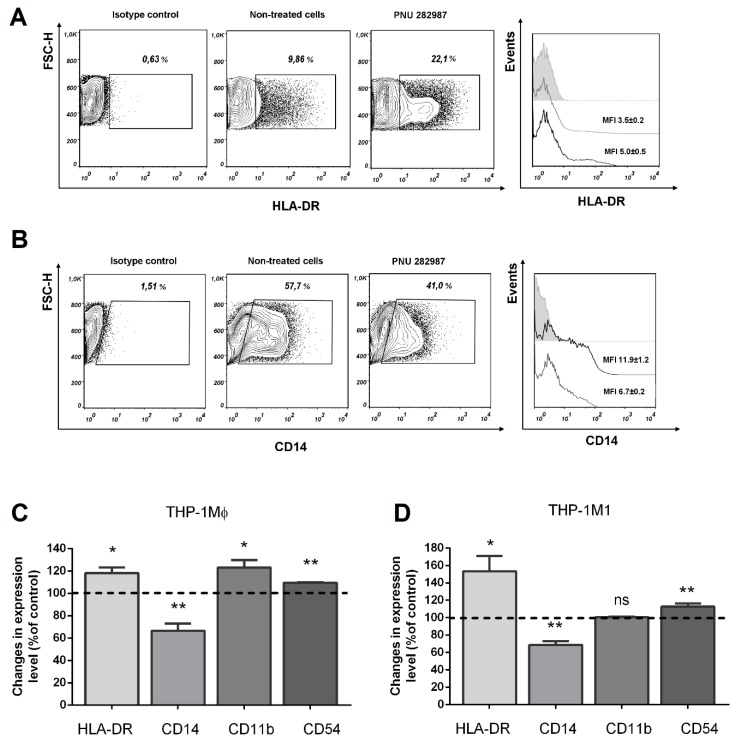
Representative expression profile of HLA-DR and CD14 on THP-1 macrophages and effects of PNU 282,987 on the expression of membrane receptors involved in the inflammation on THP-1Mϕ and THP-1M1 cells. Cells were treated with PNU 282,987 for 48 h and analyzed by flow cytometry. PNU 282,987 led to an (**A**) increase in the proportion of fluorescence and the number of HLA-DR positive cells with (**B**) a decrease in CD14 expression. Histograms: gray dashed—isotype control; black line—control cells; gray line—PNU 282,987 treated cells. The numbers in the histograms represent MFI ± SEM (geometric mean in the entire cell population). The changes were observed in the expression level of the membrane proteins HLA-DR, CD14, CD11b, and CD54 on (**C**) THP-1Mϕ and on (**D**) THP-1M1. Stimulation of cells by PNU 282,987 also led to the increased expression of CD54 cell adhesion molecules and of the complement receptor CD11b. Data were obtained in three independent experiments and presented as mean ± SEM. Student’s *t*-test: * *p* < 0.05, ** *p* < 0.01 and ^ns^ *p* > 0.05 compared to untreated control cells.

We further examined whether the α7 nAChR activation influenced the expression of such membrane molecules and receptors as HLA-DR and CD14 involved in inflammation, as well as CD11b and CD54 involved in intercellular interactions. THP-1 monocytes or primary human monocytes were differentiated to macrophages, after which THP-1 macrophages were polarized into classically activated macrophages (M1) by IFN-γ (20 ng/mL) and *E. coli* LPS (100 ng/mL). The effect of the selective α7 receptor agonist PNU 282,987 on the expression of membrane markers HLA-DR, CD14, CD54, and CD11b on THP-1Mϕ, THP-1M1 or MDMs cells was studied after 48 h incubation (Figure 5).

The cell treatment by PNU 282,987 induced a 20% enhancement in HLA-DR expression during the maturation of THP-1Mϕ (Figure 5C). This agonist induced a marked (on overage by 50%) increase in the expression of HLA-DR molecules on classically activated THP-1M1 cells (Figure 5A,D).

The action of PNU 282,987 on the THP-1Mϕ and THP-1M1 cells resulted in the inhibition of the CD14 receptor expression (Figure 5B–D), as reflected by a decrease in the number of CD14 positive cells and a lower intensity of fluorescence of these cells in comparison with the untreated control cells.

Moreover, PNU 282,987 increased the expression of macrophage adhesion molecules CD54 and of the CD11b complement receptor (Figure 5C,D), where the highest expression level was observed in THP-1Mϕ during macrophage maturation. No change in the expression of CD11b receptor in THP-1M1 in the presence of PNU 282,987 was observed.

**Figure 6:**
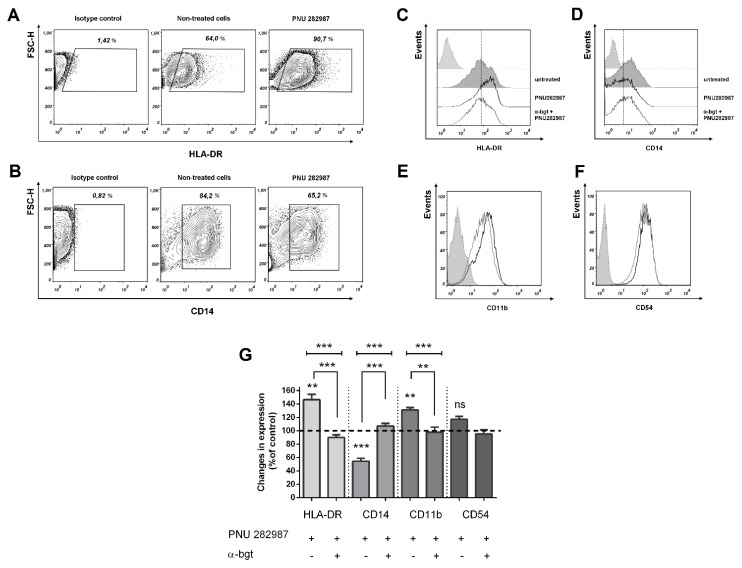
Representative expression profile and effect of PNU 282,987 on the expression of HLA-DR, CD14, CD54, and CD11b in the MDMs. The expression levels (**A**) of HLA-DR and (**B**) CD14 on the PNU 282,987 treated and untreated MDMs are shown. These cells were treated with PNU 282,987 or PNU 282,987 + α-bgt for 48 h and analyzed by Fluorescence-Activated Cell Sorting (FACS). PNU 282,987 induced an increase in the expression (**C)** of HLA-DR molecules, (**E**) CD11b, (**F**) CD54, and decreased the expression (**D**) of CD14; the effect of PNU 282,987 is blocked by the α7 receptor antagonist α-bgt. Histograms: gray dashed—isotype control; gray line—cells control; black line—PNU 282,987 treated cells. (**G**) Changes in the expression level of membrane proteins involved in inflammation on MDMs treated with PNU 282,987. The bars are expressed as mean ± SEM. The results were obtained in three experiments on MDMs from three different donors. One-way ANOVA tests with Turkey post hoc test: PNU 282,987 vs. non-treated cells and PNU 282,987 vs. PNU 282,987 + α-bgt: ** *p* ≤ 0.01, *** *p* < 0.001, ^ns^ *p* > 0.05.

The influence of PNU 282,987 on the expression of the above-listed membrane proteins was also evaluated using the MDMs: an increase in the HLA-DR (Figure 6A) and a decrease in the CD14 expression (Figure 6B) were observed. Similar changes were found with THP-1Mϕ and THP-1M1 cells.

The addition of PNU 282,987 also induced an increase in the fluorescence intensity proportion of CD11b and CD54 positive cells (Figure 6E,F). The percentage of expression of these molecules corresponded to the expression level of CD54 and CD11b on the PNU 282,987 treated THP-1Mϕ. To confirm that the upregulation of HLA-DR and decreased expression of CD14 is the result of the α7 nAChR stimulation, MDMs were preincubated with an α7 receptor antagonist α-bgt (10 µM), which abrogated the PNU 282,987-induced upregulation of HLA-DR (Figure 6C,G), inhibition of the CD14 expression (Figure 6D,G), as well as increased CD11b (Figure 6G).

### 3.4. Analysis of TNF-α, IL-6, and IL-10 Production during Activation of α7 nAChR

**Figure 7:**
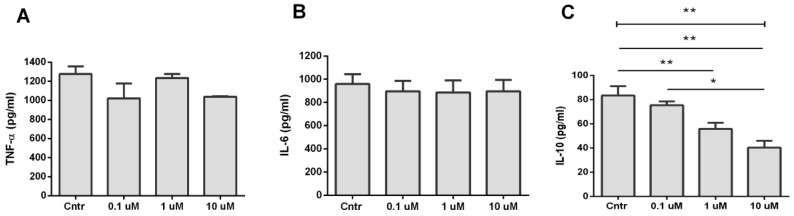
Cytokine levels in macrophage culture medium. Macrophages THP-1Mϕ were treated with various doses of PNU 282,987 for 30 min, after which they were stimulated with lipopolysaccharide (LPS). The levels (**A**) of tumor necrosis factor-α (TNF-α), (**B**) interleukin (IL)-6 and (**C**) IL-10 were determined after 24 h by ELISA. Bars are expressed as mean ± SEM. Data were obtained in three independent experiments. One-way ANOVA tests with Turkey post hoc test: * *p* < 0.05, ** *p* < 0.01.

Cytokine production during activation of α7 nAChR by PNU 282,987 was studied in LPS-stimulated THP-1 macrophages. The cells were pretreated with PNU 282,987 (0.1–10 µM) for 30 min and then stimulated with LPS (500 ng/mL) for 24 h. Such cell treatment did not significantly change the TNF-α production (Figure 7A). The α7 nAChR activation with PNU 282,987 had no effect on the IL-6 release at any of the tested concentrations (Figure 7B). However, we found a dose-dependent inhibition of the LPS-induced release of IL-10 in the PNU 282,987 treated macrophages (Figure 7C).

## 4. Discussion

The vagus nerve (cranial nerve X) is the longest of the cranial nerves, and its main function is to regulate the parasympathetic nervous system. It has been earlier shown that the vagus nerve stimulation can reduce inflammation both in the peripheral lymphoid organs and in the brain [39,40]. The complex interactions between the nervous and immune systems that make up this “inflammatory reflex” are still not fully understood [41]. However, it is clear that the anti-inflammatory effects observed after vagal stimulation are mediated by the activation of α7 nAChRs that were detected on the innate immunity cells [3].

Here we show for the first time that macrophages derived from the THP-1 cells express functional α7 nAChRs. A number of studies have found that monocytes [8], dendritic cells [42], natural killer (NK) cells [43], T and B lymphocytes [44,45] express nAChRs. We were not able to detect significant changes in calcium rise during the application of nicotine, non-selective nAChR agonist, to THP-1Mϕ. However, the use of a positive allosteric modulator (PNU 120,596) led to an increase in calcium rise when co-applied with nicotine. We showed that the specific activation of α7 nAChR mediates the transmission of Ca^2+^ signals in the THP-1Mϕ. A selective α7 nAChR agonist PNU 282,987 induced a similar increase in calcium rise as epibatidine. PNU 282,987 together with PNU 120,596 caused a steady increase in [Ca^2+^]i. α-bgt and MLA almost completely abolished the observed calcium rise in nicotine- or PNU 282,987-responsive cells. A similar type of response was recorded in other human cells, such as NK cells [43], endothelial cells [46], and peripheral blood lymphocytes [47]. In previous studies using alveolar macrophages [30], the U937 monocytic cell line [31], and mouse microglia [32], currents were not detected in response to such agonists as nicotine or acetylcholine. Using patch-clamp electrophysiology experiments, by applying PNU 282,987 or PNU 282,987 in the presence of positive allosteric modulator (PAM, PNU 120596), we confirmed that the α7 nAChRs on the THP-1Mϕ are functioning as an ion channel (see Figure 3). Similar results were previously described using MDMs when co-applying acetylcholine with PNU 120,596 [38]. The RT-PCR experiments using THP-1Mϕ cells and MDMs revealed the expression of α2, α4, α7, β3, and β4 subunits, but not of α3, α9 and β2 subunits. Padilla et al. [48] showed that the THP-1 macrophages are stained with fluorescent derivative α-bgt. The expression of α7 nAChR on the THP-1Mϕ membrane and MDMs was confirmed by cytochemical staining with a fluorescent derivative of α-bgt (AF647-α-bgt). The highest AF647-α-bgt binding to the α7 receptor was observed on the THP-1Mϕ cells. Human cell lines are important in vitro tools for studying cellular functions and the signaling pathways. Thus, macrophages derived from THP-1 cells, which express functional α7 nAChR, can serve as a convenient in vitro model for studying the role of α7 nAChR in various immunopathological processes.

The CD14 membrane protein, together with TLR4 and the MD2 adapter molecules, serves as the receptor for such components of gram-negative bacteria as LPS [49]. Binding of LPS to CD14 leads to the activation of immune cells and excessive production of TNF-α, playing a critical role in sepsis [50]. Systemic inhibition of CD14 reduces inflammation in sepsis [51]. We have found that PNU 282,987 inhibited the expression of CD14. Similar results were obtained in experiments with the THP-1Mϕ and MDMs. At the early stage of sepsis, macrophages undergo differentiation according to the classical type (M1), releasing an excessive amount of pro-inflammatory cytokines [52]. Noteworthy, we also observed a decrease in the CD14 expression on the LPS-activated THP-1M1 macrophages. The specificity of the CD14 inhibition on MDMs through the α7 receptors was confirmed by pretreatment of cells with α-bgt, an α7 nAChR antagonist. This compound reversed the effect of PNU 282,987 on the α7 receptors, returning the CD14 expression to the baseline. As a result, regulation of CD14 expression during differentiation and on activated macrophages is possible through stimulation of α7 nAChR.

A decreased HLA-DR expression on blood monocytes and macrophages occurs in the course of acute clinical inflammation, especially sepsis [53], and can contribute to the development of secondary infections. In addition, HLA-DR levels are inversely correlated with the severity of sepsis and immune dysfunction [54]. Our work has shown that PNU 282,987 promotes a higher level of expression of such MHC II molecules as HLA-DR, as compared to untreated cells. α-bgt antagonist abrogated the effect of PNU 282,987 on MDMs, supporting the α7-mediated pathway for regulating the HLA-DR expression. In particular, PNU 282,987 also increased the number and fluorescence levels of HLA-DR positive cells in the LPS-activated THP-1 macrophages, indicating that the activation of α7 receptors is effective in the process of differentiation and maturation of macrophages, as well as in their stimulation by LPS.

Adhesion molecules are known to mediate intercellular interactions, especially between the T cells and antigen-presenting cells. Lebedeva et al. [55] identified CD54 (intercellular adhesion molecule 1, ICAM-1) as a costimulatory ligand that binds to antigen-1 (lymphocyte function-associated antigen 1, LFA-1), becoming an important molecule for the activation of various T-cell populations and facilitating antigen presentation. We found that stimulation of α7 receptors on THP-1Mϕ by PNU 282,987 leads to increased expression of CD54 cell adhesion molecules. The complement system is an important component of both innate and acquired immunity, which is necessary for the recognition and elimination of pathogenic agents. We have shown that stimulation of α7 nAChR also leads to the increased expression of the CD11b receptor, which is a component of CR3 (complement receptor 3). Recent studies have reported the effects of nicotine (a non-selective nAChR agonist) on dendritic cells (DC) [56,57], showing that nicotine treatment promoted the differentiation of DC precursors in immature DC (imDC) with a semi-mature phenotype and with a higher expression of CD11c. Low doses of nicotine enhanced the expression of costimulatory molecules CD80, CD40, CD54 and HLA-DR in imDC. DCs are professional antigen-presenting cells [58] that play an important role in the aberrant immune response in sepsis [59,60]. Thus, through the activation of α7 nAChR it becomes possible to regulate several membrane markers involved in the T-cell interaction and antigen presentation.

The second part of our work concerns the cytokines. Among them, IL-10 is a pleiotropic cytokine with both anti-inflammatory and immunosuppressive properties [61]. In addition to the «cytokine storm» phase, which is characterized by the production of a large number of pro-inflammatory cytokines, the pathogenesis of sepsis is characterized by a phase of immune dysfunction. There is enough evidence that IL-10 is one of the main cytokines involved in immune dysfunction in sepsis [62,63,64,65]. Monocytes stimulated by LPS have been shown to produce high levels of pro-inflammatory cytokines IL-1, IL-6, IL-8, TNF-α, GM-CSF, and G-CSF, which can be detected within 4-8 h [66]. The production of IL-10, which has a strong anti-inflammatory effect on monocytes and Th1 lymphocytes, reaches a peak 24–48 h after stimulation. The release of IL-10 is important to suppress the inflammatory response. However, monocytes that have been re-stimulated with LPS produce low levels of pro-inflammatory cytokines, while the release of anti-inflammatory mediators, such as IL-10, increases. This «desensitization of LPS», which is mediated by IL-10, is another counter-regulatory mechanism designed to control the inflammatory response [67]. It has been suggested that monocyte deactivation that occurs during sepsis is caused by IL-10 [68]. IL-10 inhibits the release of pro-inflammatory cytokines [66], inhibits the presentation of antigen and the formation of free oxygen radicals [69]. In a series of 32 patients undergoing major abdominal surgery, IL-10 gene expression was inversely correlated with HLA-DR monocyte expression, which is consistent with the role of IL-10 as a mediator of immunosuppression [68]. Its activation (upregulation) can lead to immune tolerance in sepsis, which is manifested by a reduced ability to protect the body from secondary infections [70]. In septic shock, an increase in IL-10 release is observed, which correlates with increased mortality and secondary infections [71]. In addition, IL-10 is involved in the suppression of HLA-DR and various costimulatory receptors [72]. We have found that PNU 282,987 dose-dependently inhibits the production of IL-10 in LPS-activated macrophages indicating the important role of α7 receptors in reducing the immunosuppressive state.

Stimulation of α7 nAChR can be achieved with agonists or positive allosteric modulators, both types of ligands being potential therapeutics [73,74]. Usually, therapeutic strategies for the treatment of sepsis are aimed at suppressing the early phase of the hyperinflammatory response [75]. However, the immunosuppression state exists simultaneously with the persistent inflammation and contributes to the development of persistent, recurrent, secondary and nosocomial infections, which lead to poorer outcomes and increased mortality [59]. While therapeutic attention has long been focused on anti-inflammatory strategies, an increased understanding of the importance of sepsis-induced immune depletion in morbidity and mortality in patients with sepsis has led to a paradigm shift in sepsis research toward strategies to enhance the immune response [76]. Thus, α7 nAChR can be an important drug target not only for inhibiting hyperinflammation, but also for immune modulation.

## 5. Conclusions

This study shows that PNU 282,987 enhances the macrophage-mediated immunity and this effect is realized through α7 nAChR. The data presented provide new information on the expression of macrophages membrane markers upon activation of α7 nAChR. PNU 282,987, a selective α7 nAChR agonist, modulates immunophenotypic changes in these antigen-presenting cells by affecting the expression of surface receptors and cytokines involved in immunosuppression and hyperinflammation. In addition, we found that the changes in the level of expression of macrophage receptors on THP-1 upon stimulation of α7 receptors, are similar to changes in MDMs. These results underscore the relevance of using macrophages derived from THP-1 cells as a model for studying the role of α7 receptors in the immune processes. A deeper understanding of the regulation of immunological markers on the cells involved in inflammation is becoming a challenging task. Thus, by inducing via the α7 nAChR activation, the up- or down- regulation of biomarkers involved in the restoration of the functions of antigen-presenting cells, and as a consequence of immune homeostasis, may become a new therapeutic strategy to prevent the development of immunosuppression.

## Figures and Tables

**Table 1:** Primer sequence of genes used for qRT-PCR.

Gene	Forward	Reverse	Sources
α1	GGCTCCGAACATGAGACCCG	GCGTGACTTTGGGAGTTCCTTT	[24]
α2	TGACCCACATGACCAAGGCCCA	TGGTGAACAGCAGGTACTCGCC	[24]
α3	CCGAGGCCCCTCTACGGT	CACACAGCTTAGTGCTTA	[24]
α4	CCTCGGCCTGTCCATCGCTCA	AAGACGGTGAGCGACAGCAGC	[24]
α7	CCCGGCAAGAGGAGTGAAAGGT	TGCAGATGATGGTGAAGACC	[24,25]
α9	AGAGCCTGTGAACACCAATGTGG	ATGACTTTCGCCACCTTCTTCC	[3]
β2	GTGTCCTTCTATTCCAAT	AATGATGAAGTCATACGT	[24]
β3	AAGGGGAACAGAAGGGACGG	GAAGCAGTACGTCGCGGACG	[24]
β4	CAACAACCTGATCCGCCCAGC	GAAGGGAAAGTACTTCACCTC	[24]
β-actin	GAGCGGGAAATCGTGCGTGACATT	GATGGAGTTGAAGGTAGTTTCGTG	[26]
